# Temporal Association Between Ischemic Muscle Perfusion Recovery and the Restoration of Muscle Contractile Function After Hindlimb Ischemia

**DOI:** 10.3389/fphys.2019.00804

**Published:** 2019-06-28

**Authors:** Emma J. Goldberg, Cameron A. Schmidt, T. D. Green, R. Karnekar, D. J. Yamaguchi, E. E. Spangenberg, Joseph M. McClung

**Affiliations:** ^1^Department of Physiology, Brody School of Medicine, East Carolina University, Greenville, NC, United States; ^2^East Carolina Diabetes and Obesity Institute, East Carolina Heart Institute, Brody School of Medicine, East Carolina University, Greenville, NC, United States; ^3^Department of Cardiovascular Sciences, Brody School of Medicine, East Carolina University, Greenville, NC, United States; ^4^Division of Surgery, Brody School of Medicine, East Carolina University, Greenville, NC, United States

**Keywords:** hindlimb ischemia, angiogenesis, peripheral arterial disease, critical limb ischemia, muscle function

## Abstract

During incomplete skeletal muscle recovery from ischemia, such as that occurs with critical limb ischemia, the temporal relationship between recovery of muscle capillary perfusion and contractile function is poorly defined. We examined this relationship in BALB/cJ mice (*N* = 24) following unilateral hindlimb ischemia (HLI), which pre-clinically mimics the myopathy observed in critical limb ischemia patients. Specifically, we examined this relationship in two phenotypically distinct muscles (i.e., “oxidative” soleus – Sol and “glycolytic” extensor digitorum longus – EDL) 14- or 56-days after HLI. Although overall limb blood flow (LDPI) reached its’ recovery peak (48% of control) by HLI d14, the capillary networks in both the Sol and EDL (whole mount confocal imaging) were disrupted and competent muscle capillary perfusion (perfused lectin^+^μm^2^/muscle μm^2^) remained reduced. Interestingly, both Sol and EDL muscles recovered their distinct capillary structures and perfusion (Con Sol; 0.056 ± 0.02 lectin^+^μm^2^/muscle μm^2^, and Con EDL; 0.039 ± 0.005 lectin^+^μm^2^/muscle μm^2^) by HLI d56 (Sol; 0.062 ± 0.011 lectin^+^μm^2^/muscle μm^2^ and EDL; 0.0035 ± 0.005 lectin^+^μm^2^/muscle μm^2^), despite no further improvement in limb blood flow (LDPI). Both muscles suffered severe myopathy, indicated by loss of dystrophin positive immunostaining and the absence of stimulation induced isometric force production at HLI d14. Dystrophin immunofluorescence returned at HLI d56, although neither myofiber CSA (μm^2^) nor isometric force production (58 and 28% sustained deficits, Sol and EDL, respectively) recovered completely in either muscle. In summary, we reveal that the temporal relationship between the restoration of muscle capillary perfusion and functional ischemic skeletal muscle regeneration favors competent muscle capillary perfusion recovery in BALB/c mice in a phenotypically non-distinct manner.

## Introduction

Peripheral arterial disease (PAD) is a medical condition caused by an occlusion in the peripheral arteries, most commonly those supplying blood to the lower extremities (Norgren et al., 2007). Dysfunctional hemodynamics in the affected limb result in intermittent, pathologic limb ischemia and severe myopathy, defined by abnormal skeletal muscle function and morphology (Regensteiner et al., 1993; Brass and Hiatt, 2000; Hiatt et al., 2015; McDermott, 2018). This myopathy is universally attributed to the completeness of perfusion reduction and little is known about inherent tissue susceptibility (Callum and Bradbury, 2000), although it is a keystone manifestation of PAD and largely predicts patient morbidity and mortality (Dolan et al., 2002; Evans et al., 2011; McDermott et al., 2011, 2012; Singh et al., 2011; Jain et al., 2012).

Arteriogenesis, myogenesis, and angiogenesis are central to restoring ischemic limb function and are believed to be tightly coordinated and temporally dependent processes (Ceafalan et al., 2014). Whether or not this temporal association varies between different myofiber phenotypes, however, is not well understood. Oxidative and glycolytic muscle fibers display well-documented differences in their capillary structures and density, which contributes to their individualized relationships with tissue perfusion and oxygen distribution (Saltin and Gollnick, 2011; Liu et al., 2012). PAD patients experience a selective degradation of fast-twitch (type II) fibers that parallels presentations of increasing disease severity (Regensteiner et al., 1993; Steinacker et al., 2000; Koutakis et al., 2014), suggesting possible fiber type specific patterns or timelines of muscle regeneration. We used BALB/c mice and hindlimb ischemia (HLI), which in combination results in tissue pathology that mimics critical limb ischemia patients, to interrogate the time-based relationship between limb blood flow recovery, skeletal muscle capillary perfusion recovery and myopathy in oxidative (soleus, Sol) and glycolytic (extensor digitorum longus, EDL) limb muscles. We hypothesized that myofiber phenotypes with well-documented anatomic differences in capillary density and contractile kinetics would possess distinctive patterns of recovery, providing insight into inherent phenotypic susceptibility to limb ischemia. Our results reveal a similar temporal recovery of Sol and EDL capillary structures and perfusion after HLI that precede muscle morphological and functional recovery.

## Materials and Methods

Comprehensive methods can be found in the Supplementary Material.

### Animals

Experiments were conducted on adult male (12–18 week old) BALB/cJ mice (*N* = 24) obtained from Jackson Laboratories (Bar Harbor, ME). BALB/cJ mice were chosen for their uniformity of pathology presentation and relevance to critical limb ischemia patients. All work was approved by the Institutional Review Committee of East Carolina University. Animal care followed the Guide for the Care and Use of Laboratory Animals, Institute of Laboratory Animal Resources, Commission on Life Sciences, National Research Council. Washington: National Academy Press, 1996.

### Hindlimb Ischemia

Acute unilateral hindlimb ischemia (HLI) was performed as previously described (Schmidt et al., 2018). Mice were sacrificed by cervical dislocation or perfusion fixation 14 or 56 days after ligation (d14–56) under ketamine (90 mg/kg bodyweight) and xylazine (10 mg/kg) anesthesia. Limb blood flow was measured by laser Doppler perfusion imaging (LDPI) using a Moor Instruments LDI2-High Resolution (830 nM) System (Moor, Axminster, United Kingdom), as previously described (Schmidt et al., 2018). Images were obtained at baseline (pre), immediately following surgery (d0) and at d7, d14, d21, and d56. Two hours prior to sacrifice, 50 μL of 1 mg/mL *Griffonia simplicolia* Isolectin-B4 (GS-IB4) DyLight 594 conjugate (Vector Labs, Burlingame, CA) was injected into the right retro-orbital sinus using a 31-guage needle.

### Perfusion Fixation

Under ketamine/xylazine anesthesia, the heart was exposed to provide access to the atria and ventricles. The right atrium was punctured and a 21-gauge needle was inserted into the left ventricle. Perfusion began with a solution containing phosphate buffered saline (PBS), 10 μg/mL sodium nitroprusside, and 0.03% heparin (Dickie et al., 2006) and continued until the liver was pale in color and all of the blood was flushed from the animal. Vessels were then briefly fixed by systemic perfusion with 4% paraformaldehyde (PFA). Following fixation, EDL and Sol were carefully dissected, tied at length and immersion fixed in 4 or 2% PFA, respectively, and placed in 1× PBS overnight.

### Whole Mount Imaging

For whole muscle imaging, muscles were permeabilized in saponin, washed in 1× PBS, and blocked in 5% goat serum + 1× PBS. Samples were incubated overnight with CD31 primary antibody (1:500 dilution). Samples were then washed in 1× PBS and incubated with AF 488 conjugated anti-rat IgG secondary antibody (1:1000 dilution, Invitrogen, Carlsbad, CA), phalloidin (1:100 dilution, Invitrogen) and Nucblue (2 drops/mL). Muscles were washed and stored in 1× PBS at 4°C.

### Histology and Immunofluorescence

Muscles were placed into 30% sucrose solution for cryoprotection before being embedded in optimal cutting temperature medium (OCT) and frozen in liquid nitrogen cooled isopentane. 10 μm sections were cut using a CM-3060S cryostat (Leica) and collected on charged glass slides. Histology was performed as previously described (Schmidt et al., 2017, 2018) and included primary antibodies for rat anti-mouse CD31 (BioRad, Hercules, CA), rabbit anti-mouse dystrophin (BioRad, Hercules, CA), rabbit-anti mouse laminin (Sigma-Aldrich, St. Louis, MO), MyHC types I (BA-D5), IIa (SC-71), and IIb (BF-F3; Developmental Studies Hybridoma Bank, University of Iowa). Samples were mounted using Vectashield hard mount medium (Vector Labs) and imaged with an Evos FL auto microscope (Thermo Fisher Scientific, Waltham, MA).

### Muscle Contractile Function

Contractile function was assessed on control and ischemic Sol and EDL muscles using an Aurora 300B-LR, as previously described (Spangenburg et al., 2008; Schmidt et al., 2018; Tarpey et al., 2018). Force frequency curves were integrated and summed over time to calculate force capacity (N^∗^s/cm^2^).

### Statistical Analyses

All outcome measures and analyses were performed by individuals blinded to ischemia or control groups. Group means were compared using multiple *t*-tests. A one-way ANOVA was used to determine differences in LDPI perfusion data between all timepoints. *P* values less than 0.05 were considered statistically significant. All statistical analysis and visualization were carried out using Graphpad Prism (Version 7.0d). Image analysis was carried out using ImageJ 1.52e. Image processing was applied uniformly across all images of comparable groups. Representative images were contrast enhanced with a 0.3%-pixel saturation threshold. Images used for quantification were not altered in any way that would affect image histograms.

## Results

### Limb Blood Flow and Competent Capillary Perfusion Recovery

**FIGURE 1:**
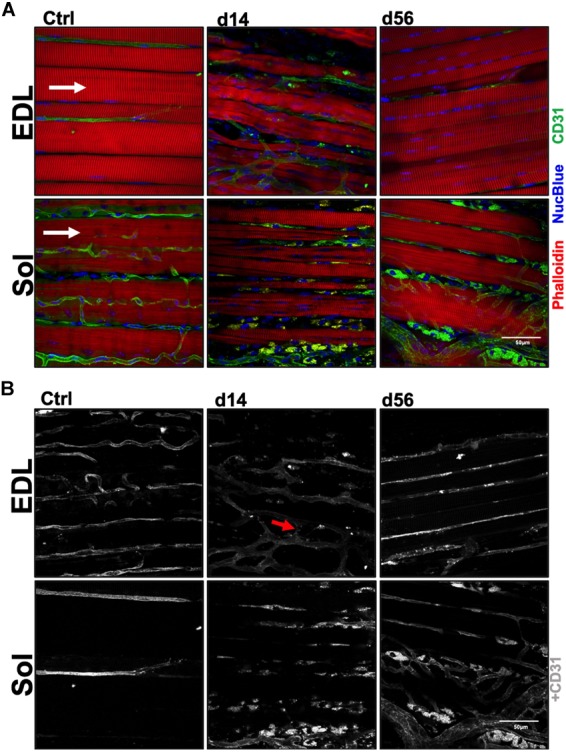
Myofiber and capillary anatomy during recovery from ischemia. **(A)** Representative 60× confocal Z-stack images of whole-Mount EDL and Sol muscles immunostained for myofiber structure (phalloidin; red), vascular structure (CD31; green) and nuclei (NucBlue; blue) in control, d14 and d56 limbs. Images qualitatively reveal the complex microenvironment within the limb following an ischemic injury and present a temporal summary of our findings. White arrows indicate myofiber direction. **(B)** Grayscale representative 60× confocal Z-stack images from panel **(A)**, presenting only CD31^+^ immunostaining. These whole-Mount EDL and Sol images specifically highlight the vascular structures in the muscles before and following ischemic injury (CD31; gray). Red arrows indicate observed sprouting angiogenesis in the ischemic microenvironment.

**FIGURE 2:**
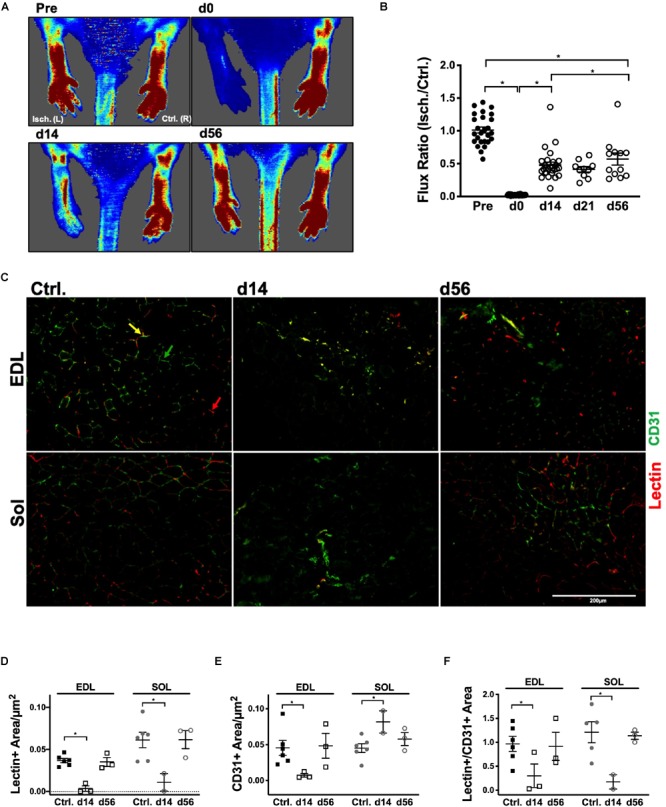
Restoration of peripheral blood flow and muscle capillary perfusion. **(A)** Representative laser Doppler perfusion images (LDPI) of the ischemic left [Isch. (L)] and control right [Ctrl. (R)] plantar paws in the prone position at baseline, d0, d14, and d56. **(B)** Quantification of flux measurements at baseline, d0, d14, d21, and d56 represented as a ratio of the left (Isch.) over right (Ctrl.) limbs. **(C)** Representative 20× immunofluorescent images of systemically DyLight 594 conjugated GS-IB_4_ lectin, which indicates vessels which were actively perfused at the time of sacrifice (red), and cluster of differentiation 31 (CD31^+^, PECAM-1; green), which indicates total endothelial cells, in transverse sections of the control, d14 and d56 Sol and EDL. Red arrows indicate lectin^+^ vessels; green arrows indicate CD31^+^ vessels and yellow arrows indicate CD31^+^/lectin^+^ vessels. **(D)** Average lectin positive area per μm^2^ in control, d14 and d56 EDL and Sol. **(E)** Average cluster of differentiation 31 (CD31), area per μm^2^ in control, d14 and d56 EDL and Sol. **(F)** Ratio of lectin positive area:CD31 positive area in control, d14 and d56 EDL and Sol. Error Bars indicate mean ± SEM. ^∗^*P* < 0.05.

Vascular structure and muscle capillary perfusion recovery were examined by employing the following: non-invasive LDPI; whole mount phalloidin and CD31^+^ immunostaining; and transverse muscle section CD31^+^ and isolectin^+^ immunostaining and quantification. 60× confocal Z-stacks provided us with qualitative information regarding anatomical muscle transformations throughout the regenerative process (Figure 1A,B). Sprouting angiogenesis was observed in both muscles at HLI d14 (indicated with red arrows in Figure 1B). By HLI d56, both muscles had re-established vascular networks with similar properties as the control muscles: i.e., winding and dense Sol capillaries and parallel organization of the EDL vasculature along the myofiber long axis. LDPI revealed a significant decrease in plantar paw blood flow at all time points following HLI induction (Figure 2A). The flux ratio of the ischemic/control limb (AU) was reduced immediately following HLI (d0) and remained attenuated through HLI d56 (d56 flux ratio mean: 0.5696 ± 0.09169) (Figure 2B). Immunohistochemistry of CD31^+^ cells and lectin^+^ vessels in transverse muscle sections (Figure 2C) reveal reduced lectin positive areas (lectin^+^μm^2^/muscle μm^2^; used as an indicator of competent capillary perfusion) in both the ischemic Sol and EDL at HLI d14 (Ctrl. Sol: 0.067 ± 0.0014, Isch. Sol: 0.01 ± 0.01, *P* = 0.006; Ctrl. EDL: 0.035 ± 0.001, Isch. EDL: 0.0034 ± 0.003 *P* < 0.001). Both Sol and EDL lectin perfusion areas were restored to contralateral baseline values by HLI d56 (Ctrl. Sol: 0.056 ± 0.02, Isch. Sol: 0.062 ± 0.011, *P* = 0.73; Ctrl. EDL: 0.039 ± 0.005, Isch. EDL: 0.035 ± 0.005 *P* = 0.841) (Figure 2D). Restoration of lectin^+^ area in transverse muscle sections indicates recovery of capillary perfusion in the muscle. CD31^+^ area (CD31^+^μm^2^/muscle μm^2^; used as an indicator of total capillary number) was significantly reduced in the EDL at HLI d14 (Ctrl. EDL: 0.046 ± 0.01, Isch. EDL: 0.007 ± 0.003 *P* = 0.041). Interestingly, CD31^+^ area was increased in the Sol at HLI d14 (Ctrl. Sol: 0.045 ± 0.006, Isch. Sol: 0.082 ± 0.015, *P* = 0.033). CD31^+^ positive area returned to control values in both the Sol and EDL by HLI d56 (Figure 2E). The ratio of lectin^+^:CD31^+^ areas (used as an indicator of competent perfused capillary vessels out of the total endothelial signal present) decreased in both the ischemic Sol and EDL at HLI d14 (Ctrl. Sol: 1.21 ± 0.49, Isch Sol: 0.17 ± 0.21 *P* = 0.04; Ctrl EDL: 0.97 ± 0.38, Isch. EDL: 0.30 ± 0.43, *P* = 0.049) but was restored to contralateral control values at HLI d56 (Figure 2F).

### Muscle Structural and Functional Recovery After HLI

**FIGURE 3:**
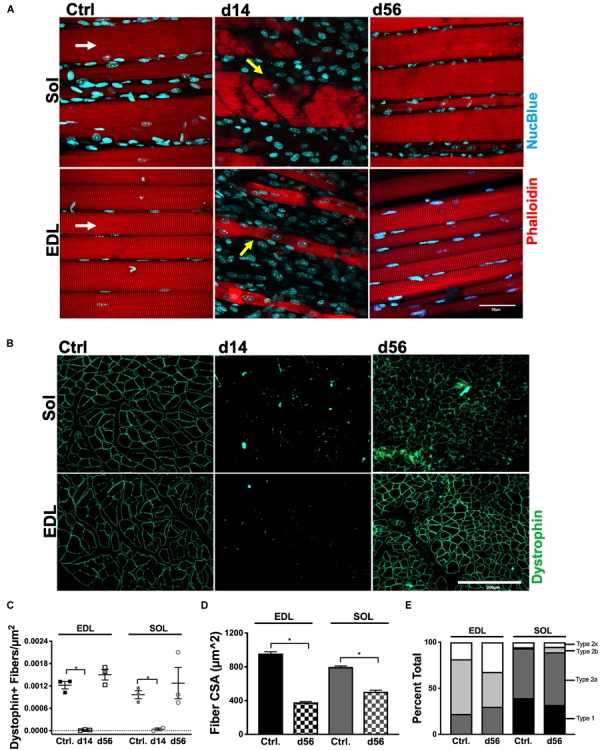
Myofiber structural recovery. **(A)** Representative 60× Z-stacks of control, d14 and d56 EDL and Sol. Myofiber structure is represented by phalloidin immunostaining (red) and counterstained with NucBlue (blue). White arrows indicate myofiber direction. Yellow arrows indicate damaged myofibers. **(B)** Representative 20× immunofluorescence images of dystrophin positive immunostaining in transverse sections of control, d14 and d56 EDL and Sol. **(C)** Average dystrophin-positive immunostained myofibers per μm^2^ in control, d14 and d56 EDL and Sol. **(D)** Average CSA (μm^2^) of myofibers in control and d56 EDL and Sol. **(E)** Fiber type distribution in control and d56 EDL and Sol **(E)**. ^∗^*P* < 0.05.

Whole mount imaging qualitatively revealed the extent of myofiber degeneration and regeneration after HLI (indicated with yellow arrows in Figure 3A). Transverse sections of the Sol and EDL (Figure 3B) were utilized to quantify the number of dystrophin^+^ fibers per μm^2^. At HLI d14, both ischemic Sol and EDL muscles suffered from significant reductions in the number of dystrophin^+^ fibers. At d56, dystrophin^+^ fiber numbers were returned to contralateral control values in both muscles (Figure 3C). Fiber cross-sectional area (CSA, μm^2^) was measured at HLI d56 and revealed attenuated myofiber sizes (CSA, μm^2^) in the ischemic Sol and EDL compared with their respective contralateral controls (Ctrl. Sol: 798.1 ± 11.9, Isch. Sol: 506.8 ± 17.76, *P* = 0.23; Ctrl. EDL: 956.6 ± 22.11, Isch. EDL: 379.9 ± 8.64, *P* = 0.016) (Figure 3D and Supplementary Figure S2A). Compared to contralateral controls, the distribution of MHC fiber types was not altered in the ischemic Sol or EDL (Figure 3E).

**FIGURE 4:**
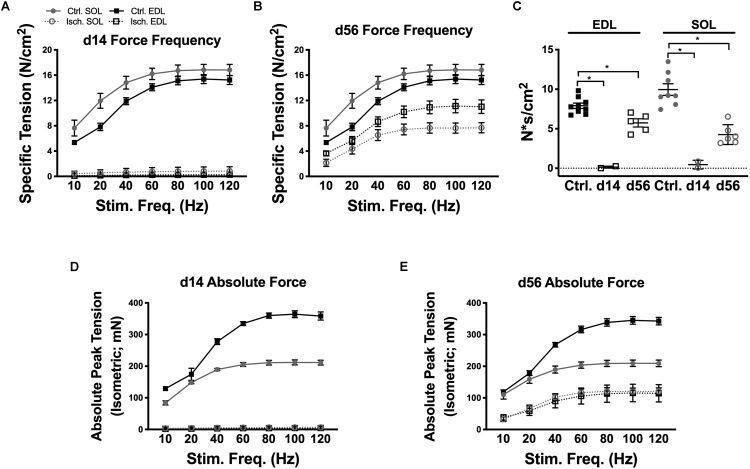
Muscle contractile function recovery. Force frequency curves for ischemic and control Sol and EDL muscles at d14 **(A)** and d56 **(B)**. **(C)** Force capacity (measured by integrating the force frequency curves and summing the values over time) of control, d14 and d56 EDL and Sol. Absolute peak isometric tension curves for ischemic and control Sol and EDL muscles at d14 **(D)** and d56 **(E)**. Bars indicate mean ± SEM. ^∗^*P* < 0.05.

Force frequency (FF) protocols were performed to measure total and specific tension in control, HLI d14 and HLI d56 EDL and Sol (Figure 4A,B). After 14d HLI, neither the Sol nor EDL were capable of measurable force production (when dystrophin immunoreactivity was largely absent). At HLI d56, the ischemic EDL was only able to produce 72% of maximal control force (100 Hz) and the ischemic Sol produced 42% of maximal control force (80 Hz). This signifies persistent deficits in muscle contractile function, which are more severe in the oxidative Sol. Force frequency curves were integrated and summed over time to calculate force capacity (N^∗^s/cm^2^). Force capacity was significantly reduced at both HLI d14 (Ctrl. Sol: 58.60 ± 5.498, Isch. Sol: 2.719 ± 2.332, *P* = 0.23; Ctrl. EDL: 40.43 ± 2.272, Isch. EDL: 0.7552 ± 0.641, *P* = 0.016) and d56 (Ctrl. Sol: 58.60 ± 5.498, Isch. Sol: 506.8 ± 3.278, *P* = 0.23; Ctrl. EDL: 40.43 ± 2.272, Isch. EDL: 29.84 ± 2.756, *P* = 0.016) (Figure 4C).

The specific force values measured in this study are consistent with values previously recorded by our lab and our collaborators using our experimental procedures (Regensteiner et al., 1993; Callum and Bradbury, 2000; Norgren et al., 2007; Hiatt et al., 2015); we have consistently measured maximum specific force of Sol and EDL muscles to be between 15 and 25 N/cm^2^. Variations in specific force values between our lab and others may be due to the inbred mouse strain utilized or the extent to which the muscle is dried prior to obtaining its wet weight. The absolute force values achieved in our experiments (Figure 4D,E), however, are similar to those previously outlined by Brooks and Faulkner (Dolan et al., 2002). Control EDL and soleus muscles isolated from the 12–18-week-old BALB/cJ mice used in this study reached maximal absolute force values of 364 and 211 mN, respectively, comparable with the 413 and 213 mN absolute force values that were recorded for 2–3-month-old C57BL/6 mice in their study (Dolan et al., 2002).

## Discussion

Effectively recovering limb skeletal muscle contractile function within an ischemic environment is dependent upon the successful reestablishment of both tissue perfusion and anatomic muscle architecture. We examined the temporal relationship between the restoration of ischemic limb blood flow, muscle capillary bed organization, competent vessel perfusion, and myofiber regeneration in physiologically distinct muscles. Our findings reveal that capillary perfusion and muscle structural organization (dystrophin) are restored within 8 weeks of ischemic onset by HLI in oxidative and glycolytic muscles of BALB/cJ mice. Despite peak restoration of overall limb blood flow and complete restoration of muscle capillary perfusion, myofiber sizes and muscle force capacity do not recover on the same timeline in either oxidative or glycolytic muscle phenotypes. Our contractile measurements were performed in a controlled bath facilitating the diffusive flux of oxygen in muscles isolated from an ischemic environment *in vivo*. Effectively, we removed any confounding influences of differential capillary perfusion in our functional force measurements. 56-days post HLI, despite quantitative restoration of competent capillary perfusion *in vivo*, muscles are severely functionally and anatomically compromised even in an environment where diffusive oxygen is readily available across the length of the muscle. Combined with the histological evaluations, we interpret this data to reveal that the observed functional deficits are likely a result of the delays in structural/anatomical repair of the muscle fibers. Overall, our data reveal that ischemic myopathy persists similarly in oxidative and glycolytic BALB/c myofibers long after ischemic onset, and that both muscle types share a similar temporal restoration of tissue perfusion.

The temporal recovery of limb blood flow from nadir is a commonly studied process pre-clinically. BALB/cJ mice, in particular, suffer incomplete limb blood flow recovery across multiple models of hindlimb ischemia (McClung et al., 2012, 2016; Hazarika et al., 2013; Dokun et al., 2014; Schmidt et al., 2017, 2018). This deficit is commonly attributed to a differential density of pre-existing intermediate collateral vessels prior to ischemia and/or reduced ability to generate new collaterals via arteriogenesis. In this study, peak recovery of limb/plantar paw blood flow, measured by LDPI, occurred within 14-days of surgically induced ischemia. The nature of the surgery used in this study (isolation and transection at the proximal end of the femoral artery) is insufficient to induce outward tissue necrosis (paw lesions or auto amputation), even across the 56d period of ischemic recovery. In this instance, the temporal restoration of peak limb/paw blood flow was disconnected from the muscle tissue specific restoration of capillary perfusion. This demonstrates that the recovery of individual muscle tissue capillary perfusion is regulated locally and is distinct from that of the limb/paw blood flow that presumably rescues toe/paw lesion formation in these mice. Given the anatomic disruption we observed in vascular structures at HLI d14, the restoration of locally competent capillaries is likely a key limiting factor in the ability of the limb to shunt any restored flow to anterior and posterior skeletal muscles. In the case of the Soleus muscle, this is uniquely defined by the maintenance of CD31^+^ signal at HLI d14, despite a lack of competent perfusion of those vessels. In the case of the EDL, this is defined by a reduction of CD31^+^ signal combined with lack of vessel perfusion.

Clinically, pathologic limb ischemia is accompanied by intermittent bouts of ischemic insult, which cause cycles of myofiber degeneration and regeneration (Chargé and Rudnicki, 2004). When successful, regenerative processes result in an innervated, vascularized, contractile skeletal muscle that is indistinguishable from its non-ischemic counterpart (Ceafalan et al., 2014; Musarò, 2014). In general, myopathic diseases are tightly paralleled with vascular network degradation. A partial explanation for the failure of therapeutic angiogenesis trials in critical limb ischemia patients may be that these therapies fail to stimulate the survival and regeneration of ischemic muscle myofibers, which face a harder path to full restoration than the capillary networks that feed them. Both muscles examined restored functional, individualized vascular networks within 56 days: the Sol remodeled its convoluted network; the EDL remodeled its myofiber-parallel network. A similar timeline of vascular function and structure recovery in these phenotypically distinct muscles suggests that local signals within each muscle are capable of guiding vascular progenitors and endothelial cells through complex and distinct organizational processes. Despite this, both muscle phenotypes continued to suffer from persistent myopathy through 56 days. This indicates that processes related to vascular reorganization are temporally prioritized over those related to contractile function restoration in the limb, but do not guarantee complete functional recovery of the affected muscles. Although the reason for the temporal prioritization of revascularization in regenerating skeletal muscle is not completely understood, a logical explanation is that sufficient blood supply is required to maintain functional skeletal muscle (Messina et al., 2007; Kärkkäinen et al., 2008; Deasy et al., 2009). Additionally, studies have highlighted a role for angiogenesis in increasing the amount of active blood-vessel related stem cells that can participate in skeletal muscle regeneration (Dellavalle et al., 2007; Zheng et al., 2007). Dynamic and reciprocal interactions between regenerating skeletal muscle and angiogenesis are accepted as pivotal components of juvenile dermatomyositis (JDM) myopathy and Duchenne muscular dystrophy (DMD) (Latroche et al., 2015) and have led to approaches to treat muscular dystrophies with phosphodiesterase-5 inhibitors like Tadalafil (Asai et al., 2007) to improve blood flow (Martin et al., 2014) and muscle regeneration. The results of this study, in a common pre-clinical model of PAD, support the development of dual therapy approaches to improve both the temporal restoration of muscle capillary perfusion and the regeneration of muscle myofibers to restore full functional capacity.

Several articles have reported the selective degradation of faster-twitch fibers during the ischemia that occurs in PAD patients (Regensteiner et al., 1993; Steinacker et al., 2000; Koutakis et al., 2014). A benefit to this adaptation would be clearly evident if slower/oxidative myofibers demonstrated a regenerative or functional advantage under the intermittent ischemic conditions caused by PAD. In this study, we took advantage of the fiber type homogeneity observed in the predominantly oxidative Sol and predominantly glycolytic EDL mouse muscles. Thus, if oxidative fibers harbored an inherent regenerative advantage, the Sol muscle should recover from ischemic injury more completely and efficiently. Despite muscle type-specific differences in vascular anatomy and contractile characteristics, the oxidative Sol and glycolytic EDL muscles appear to possess similar angiogenic timelines in our model system. It is important to note the context of the studies performed here when interpreting this finding. Mice of the BALB/c parental strain most accurately mirror the myopathy and vasculopathy seen in critical limb ischemia patients in the pre-clinical HLI model (McClung et al., 2012, 2016; Hazarika et al., 2013; Dokun et al., 2014; Schmidt et al., 2017, 2018). PAD presents as either intermittent claudication (IC; pain with exertion that is relieved with rest) or critical limb ischemia (CLI; pain at rest with or without tissue necrosis or gangrene). Although less common than IC, CLI carries a substantially higher morbidity and mortality; CLI patients have a risk of major amputation or death that approaches 40% in 1 year (Hirsch et al., 2001; Taylor et al., 2009). Very little is known about the biology of limb skeletal muscle in CLI patients. Recently, we observed no change in myofiber phenotype in the gastrocnemius muscles of CLI patients (Ryan et al., 2018). CLI patients are largely intolerant to exercise and suffer high rates of morbidity, even after surgical interventions that restore blood flow to the affected limb. This clinical disconnect parallels the temporal disconnect we observed between muscle perfusion reconciliation and persistent myopathy in BALB/c muscles.

## Conclusion

The timeline required to regenerate a fully functional skeletal muscle is dependent upon the concomitance of both angiogenic and myogenic processes. As we have shown, these processes do not necessarily reach completion within the same timeline. Furthermore, the restoration of tissue perfusion does not guarantee timely muscle functional recovery. Our results may partially explain why, despite a marked increase in the number of lower extremity revascularization procedures, functionality tied to morbidity and mortality in the PAD patient population remains largely unchanged (Goodney et al., 1946; Sampson et al., 2014). A more precise understanding of the persistent myopathy in the presence of restored limb blood flow and tissue perfusion provides an opportunity to develop adjuvant therapies to better ensure the success of surgical revascularization procedures and potentially identify patients most at risk for severe manifestations of PAD and other myodegenerative diseases.

## Data Availability

The datasets generated for this study are available on request to the corresponding author.

## Ethics Statement

All work was approved by the Institutional Review Committee of East Carolina University. Animal care followed the Guide for the Care and Use of Laboratory Animals, Institute of Laboratory Animal Resources, Commission on Life Sciences, National Research Council. Washington: National Academy Press, 1996.

## Author Contributions

EG and CS contributed to experimental design, data collection, analysis, and manuscript preparation. TG contributed to data collection, analysis, and manuscript preparation. RK contributed to data collection and manuscript preparation. DY contributed to data analysis and manuscript preparation. ES contributed to experimental design, data analysis, and manuscript preparation. JM served in conceptualization, experimental design, funding of the project, data collection, analysis, and manuscript preparation.

## Conflict of Interest Statement

The authors declare that the research was conducted in the absence of any commercial or financial relationships that could be construed as a potential conflict of interest.
