# Supplementary figures and images for: Temporal Association Between Ischemic Muscle Perfusion Recovery and the Restoration of Muscle Contractile Function After Hindlimb Ischemia
Source: Front Physiol. 2019 Jun 28;10:804. doi: 10.3389/fphys.2019.00804 (PMC6611152; doi:10.3389/fphys.2019.00804)

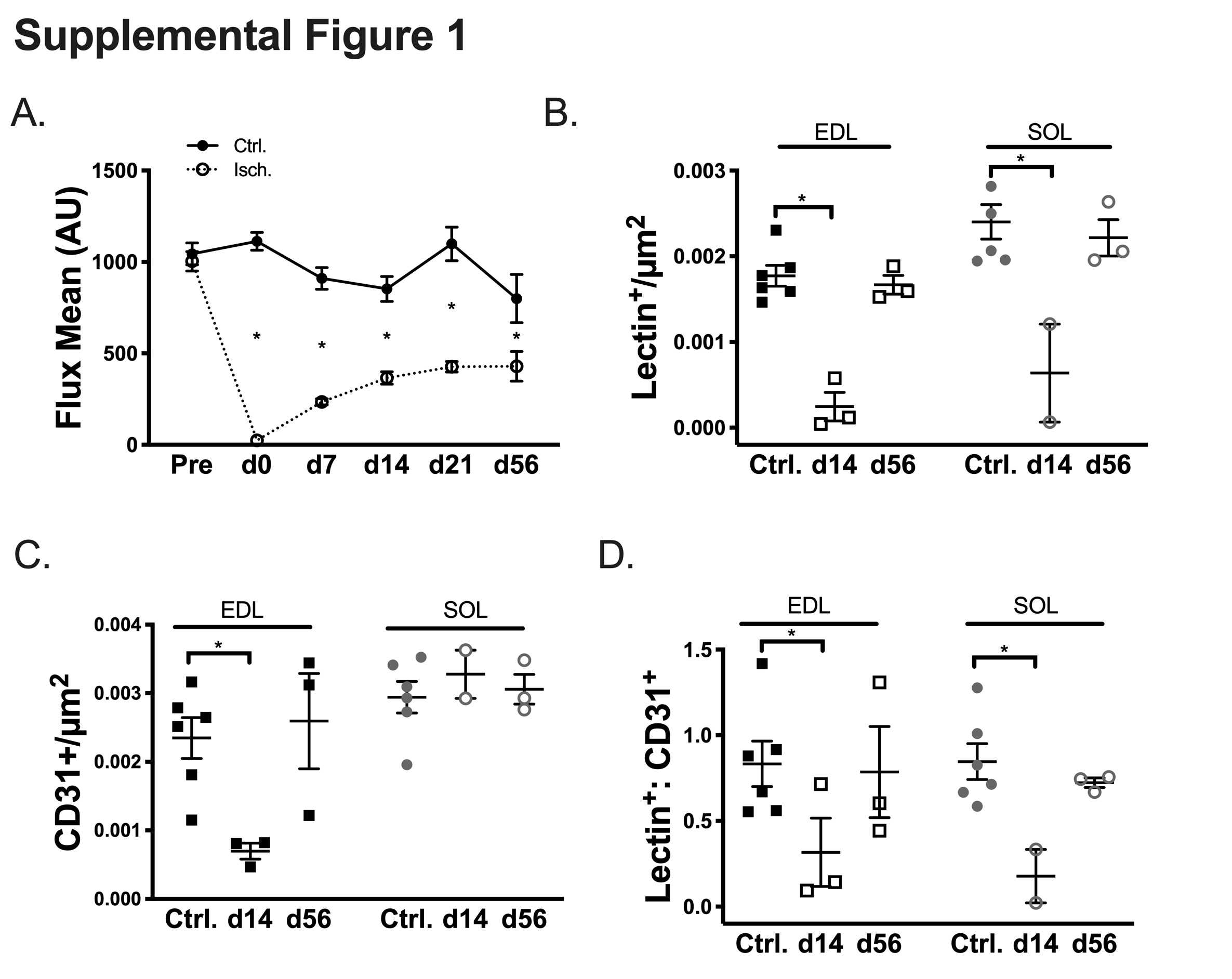

Supplement: Supplementary file 1 [file Image_1.JPEG]

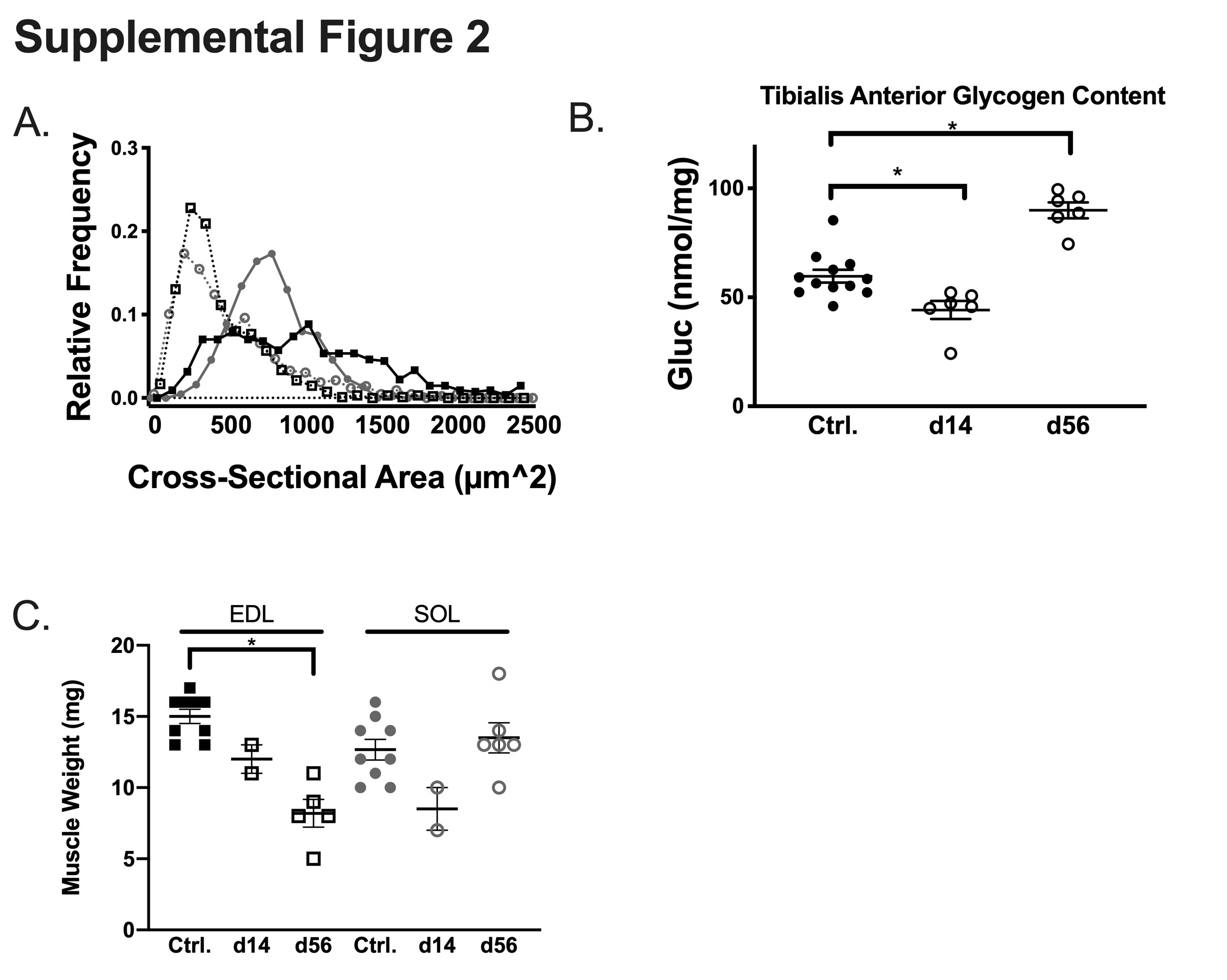

Supplement: Supplementary file 2 [file Image_2.JPEG]
