# Supplementary material for: Temporal Association Between Ischemic Muscle Perfusion Recovery and the Restoration of Muscle Contractile Function After Hindlimb Ischemia
Source: Front Physiol. 2019 Jun 28;10:804. doi: 10.3389/fphys.2019.00804 (PMC6611152; doi:10.3389/fphys.2019.00804)
Supplement: Supplementary file 3 [file Data_Sheet_1.PDF]

Temporal association between ischemic muscle perfusion recovery and the restoration of muscle contractile function after hindlimb ischemia: Supplemental Material

Running title: Fiber type and ischemic functional recovery

Supplemental Material

Emma J. Goldberg[1,2], Cameron A. Schmidt[1,2], T.D. Green[1,2], R. Karnekar[1,2], D.J. Yamaguchi[3,4], E.E. Spangenberg[1,2], & Joseph M. McClung[1,2,3*]

[1]Dept. of Physiology, Brody School of Medicine; [2]East Carolina Diabetes and Obesity Institute; [3]Department of Cardiovascular Sciences, [4]Division of Surgery, Brody School of Medicine, East Carolina University, Greenville NC

*Correspondence should be addressed to J.M.M.: Diabetes and Obesity Institute, Office #4109, Mail Stop 743, East Carolina Heart Institute, Brody School of Medicine at East Carolina University, 115 Heart Drive, Greenville, NC 27834-4354. Tel: 252-737-5034 (office); Fax: 252-744-3460; email: mcclungj@ecu.edu

Text : 1984 Pages: 14; # Tables: 0; # Figures: 4; # Supplementary Figures: 2

This work was made possible by a grant from the NIH to JMM (R01HL125695).

Supplementary Material:

Methods:

Model of Hindlimb Ischemia

Acute unilateral hindlimb ischemia (HLI) was induced as previously described[1]. Mice were anesthetized by intraperitoneal injection of ketamine (90mg/kg bodyweight) and xylazine (10mg/kg). A small (<4mm) incision was made between the left inguinal fat pad and the peritoneum. The femoral artery was carefully located from the femoral vein distal to the inguinal ligament and proximal to the lateral circumflex branch of the femoral artery. Two ligatures were placed at each end of the isolated region and the femoral artery was transected between them. Subcutaneous buprenorphine (.5mg/kg) was administered for pain management during recovery. Contralateral legs were left intact and were used to produce internal (non-ischemic) control muscles. All surgeries were performed by an experienced surgeon to minimize technical variation. Surgeries were performed within the first six hours of the animals' 12-hour light cycle. All animals had free access to food and water prior to and following surgery. Body temperature of about 37C was maintained during recovery using a heated circulating water pad. Mice were sacrificed at either 14 or 56 days after ligation (d14-56) by cervical dislocation under ketamine/xylazine, or by perfusion fixation under ketamine/xylazine.

Blood Perfusion

Limb blood flow was measured using laser Doppler perfusion imaging (LDPI), as previously described[1]. Briefly, animals were anesthetized with ketamine/xylazine and placed on a 37C warming pad in a windowless room with controlled lighting. Imaging was performed on the plantar paws at 4ms/pixel scan rate using a Moor Instruments LDI2-High Resolution System (830nm) (Moor, Axminster, UK). Hindlimb hair was removed with a microshaver (Wahl, Sterling, Illinois) prior to scanning. Laser Doppler images were obtained prior to surgery (baseline) immediately following surgery (d0) and at d7, d14, d21 and d56. Images were analyzed with the MoorLDI Image Review software.

Perfused Vessel Labeling

Two hours prior to sacrifice, 50uL of 1mg/mL *Griffonia simplicolia* Isolectin-B4 (GS-IB4) Dylight594 conjugate (Vector Labs, Burlingame, CA) was injected into the right retro-orbital sinus using a 31-guage needle. For epifluorescence microscopy in transverse and longitudinal sections, Sol and EDL muscles were isolated from control and ischemic limbs immediately following perfusion fixation.

Whole Mount Muscle Imaging

Following overnight incubation in 1X PBS, whole muscles were permeabilized in 500uL of 30ug/mL saponin for 10 minutes. Muscles were then washed in PBS 3X for 5 minutes and blocked in 5% goat serum + 1X PBS for one hour at 4C rotating end over end. Whole muscles were then incubated with rat

anti-mouse CD31 (PECAM-1) at 1:500 overnight at 4C rotating end-over-end. Following primary incubation, muscles were washed for 3X 5 minutes in 1X PBS and incubated with goat anti-rat AF 488 at 1:1000, phalloidin at 1:100 and Nucblue at 2 drops/mL at 4C for 1 hour rotating end-over-end. Muscles were washed 3X for 5 minutes with 1X PBS and stored in 1X PBS at 4C until imaging. Whole mount muscle Z-stack images were taken at proximal, midbelly and distal regions along the muscle using an Olympus FV1000 laser scanning confocal microscope with Fluoview FSW acquisition software version 4.2 (Olympus, Tokyo, Japan). A 60X plan apochromatic oil immersion objective lens was used for all representative imaging (NA 1.35). Excitation of DAPI and Alexa Fluor 488 was achieved using the 405 nm and 488 nm line of a multiline argon laser, respectively. Alexa Fluor 594 (phalloidin) excitation was achieved using a 559 nm laser diode. Images were sequentially scanned at 2 μs/pixel using Kalman line filtering. Image processing was performed using ImageJ (NIH, v1.52e). All processing was performed uniformly over the entire image, and processing parameters were made constant for the entire image set. Representative maximal intensity Z projections from whole mount muscles were contrast enhanced to facilitate feature enhancement in representative images only. No images used for quantification were altered from their original format prior to analysis.

Transverse Sections

Following overnight incubation in 1X PBS, muscles were placed into 30% sucrose solution for cryoprotection prior to being embedded in optimal cutting temperature medium (OCT) and frozen in liquid nitrogen cooled isopentane. 10um sections were cut using a CM-3060S cryostat (Leica, Buffalo Grove, IL) and collected on charged glass slides. Sections were blocked in 5% goat serum + 1X PBS for one hour at room temperature. Following blocking, sections were incubated with rat anti-mouse CD31 (PECAM-1), to counterstain for total vessels (1:100 dilution, BioRad, Hercules, CA), and rabbit anti-mouse dystrophin (1:100 dilution, BioRad, Hercules, CA), to stain for fiber structure, primary antibodies overnight at 4C. Sections were washed 3X for 5 minutes each with 1X PBS at room temperature and incubated with goat anti-rat AF 488 and goat anti-rabbit AF647 secondary antibodies (1:250, Invitrogen, Carlsbad, CA) and NucBlue (2 drops/mL) for 1 hour at room temperature. Sections were mounted using Vectashield hard mount medium (Vector Labs) and imaged with an Evos FL auto microscope (Thermo Fisher, Waltham, MA) with a plan fluorite 10 X cover slip corrected objective lens (NA = .25, air) and 20X cover slip corrected objective lens (NA = .5, air). The following excitation/emission filter cubes were used: DAPI (357/44 nm Excitation; 447/60 nm Emission), GFP (470/22 nm Excitation; 510/42 nm Emission), Texas Red (585/29 nm Excitation; 624/40 nm Emission), and Cy5 (628/40 nm Excitation; 692/40 nm Emission). One 10X and three 20X images were acquired from each sample. 10X images were used to quantify lectin positive and 20X images were used to quantify dystrophin positive fibers and

fiber cross-sectional area. 20X images were used to quantify CD31 positive signal. Lectin positive area was quantified by decomposing 10X images into red/blue/green composites, splitting channels, setting appropriate standard threshold value limits, subtracting average background values and measuring the percent area of Dylight594 (red) positive signal within each image. Image processing was performed using ImageJ (NIH, v1.52e.) All processing was performed uniformly over the entire image, and processing parameters were made constant for the entire image set. No images used for quantification were altered from their original format prior to analysis. All images were thresholded using the Yen function within ImageJ. Thresholded area and the number of particles analyzed were used as measures of perfused vessel area and number of perfused vessels. Technical replicates were averaged, and data are represented by mean fluorescence positive area and as a ratio of the ischemic to the control limb. Dystrophin positive fibers were counted over three 20X images and used as a measure of intact muscle fibers per muscle area.

Glycogen Assay

Control and ischemic anterior (TA) muscles and liver tissues were flash frozen in liquid nitrogen and stored at -80C. Glycogen assays were performed using acid hydrolysis and an enzyme coupled assay[13]. Briefly, tissue samples were digested/hydrolyzed under harsh acidic conditions using 2N hydrochloric acid (Sigma Aldrich, St. Louis, MO) on a heating block at 95C for 2 hours with additional vortexing. Samples were neutralized with equal volume 2N sodium hydroxide (Sigma). A small amount of tris HCl pH 7.0 (~1% of final volume) was added to buffer the solution. Samples were added to a clear 96 well plate in duplicate and were incubated with hexokinase reagent (Thermo Fisher) or water for background correction. A standard curve of D-glucose was used to calculate the concentrations of hydrolyzed glucosyl units in each sample. Colorimetric measurement of NADH absorbance was made at 340nm using a Cytation 5 microtiter plate reader (Biotek, Winooski, VT).

Muscle Contractile Function

Extensor Digitorum Longus (EDL) and Soleus (Sol) muscles were surgically excised from anesthetized mice with 5-0 silk sutures at the proximal and distal tendon ends. Whole EDL and Sol muscles were mounted in a horizontal, controlled bath at 25C. One end of the muscle attached to a fixed post and the other end attached to a force transducer (Aurora 300B-LR, Aurora, ON, Canada) operated in isometric mode. The bath contained modified Kreb's buffer solution (PSS; pH 7.2) containing 115mM NaCl, 2.5mM KCl, 1.8mM $CaCl_2$, 2.15mM Na_2HPO_4, .85mM NaH_2PO_4 and was continuously aerated with 95% O_2/5% CO_2 throughout the experiment. Muscles were then allowed to equilibrate in the bath for 10 minutes. Following the equilibration period, muscle tension was optimized by subjecting the muscles to a supramaximal

voltage and adjusting muscle length until peak twitch contraction force was achieved. Force frequency curves were generated for each muscle by delivering 200ms pulse trains at increasing frequencies of 10, 20, 40, 60, 80, 100, and 120 Hz. Following the experimental protocol, the muscle length was determined with a digital micro-caliper. Muscles were then trimmed proximal to the suture connections, blotted to remove excess moisture, and wet weights were obtained. The cross-sectional area for each muscle was determined by dividing the mass of the muscle (g) by the product of its length (L_0, cm) and estimated muscle density (1.06 g cm^{-3}). Muscle force production is expressed as specific force (N/cm^2), which is determined by dividing the tension (N) by the calculated muscle cross-sectional area. Representative 60X confocal Z-stack images of whole-Mount EDL and Sol muscles.

The shapes of the force frequency curves recorded for the fast- contracting EDL and slow-contracting Sol in this study are consistent with the contractile properties of fast and slow twitch muscles, respectively[7]. The EDL muscle produces a greater absolute force compared with the soleus. Additionally, the soleus reaches its peak force at a lower stimulation frequency (60-80Hz) compared with the EDL (100-120Hz). Although the specific force frequency curves produced in this experiment may not appear to possess markedly different shapes, they are consistent with force frequency curves previously published by our group and others. Similarities between the curve shapes in the specific force frequency graphs could be due to the parental inbred mouse strain tested (BALB/cJ), or effects seen in the contralateral limb in the weeks following HLI induction. The differeneces in curve shapes between the two muscles are exaggerated in the graphs of absolute force, where the force values attained align with those put forth by Brooks and Faulkner[5]. Overall, our data indicate a significant deficit in the contractile ability of the ischemic Sol and EDL muscles when compared with their internal, contralateral controls, irrespective of curve shape.

Supplementary Figure Legends:

SF1: (A) Flux mean (AU) in control and ischemic prone paws at pre-HLI, immediately post-HLI (d0) and at d7-d56. (B) Average lectin positive particles per transverse CSA in control, d14 and d56 EDL and Sol. (C) Average CD31 positive particles per transverse CSA in control, d14 and d56 EDL and Sol. (D) Ratio of lectin positive particles:CD31 positive particles in control, d14 and d56 EDL and Sol. Bars indicate mean ± SEM. *$P<0.05$.

SF2: (A) Relative frequency distribution of CSA in control and HLI d56 EDL and Sol. (B) Glycogen content in control, d14 and d56 tibialis anterior (TA) muscle tissue (F). Weights of control, d14 and d56 EDL and Sol muscles (C). Bars represent mean ± SEM.

1. Schmidt, C. A. *et al.* Strain-Dependent Variation in Acute Ischemic Muscle Injury. *Am. J. Pathol.* **188**, 1246–1262 (2018).
2. Ourdé, C. H., Ignaud, A. V, Eurdy, I. B., Artelly, I. M. & Eller, A. K. Sustained Peripheral Arterial Insufficiency Durably Impairs Normal and Regenerating Skeletal Muscle Function. **56**, 361–367 (2006).
3. Iyer, S. R., Valencia, A. P., Hernández-ochoa, E. O. & Lovering, R. M. Skeletal Muscle Regeneration in the Mouse. **1460**, 293–307 (2016).
4. Tarpey, M. D. *et al.* Characterization and utilization of the flexor digitorum brevis for assessing skeletal muscle function. *Skelet. Muscle* **8**, 1–15 (2018).
5. Tarpey, M. D. *et al.* Induced in vivo knockdown of the Brca1 gene in skeletal muscle results in skeletal muscle weakness. *J. Physiol.* **597**, 869–887 (2019).
6. Collins, B. C. *et al.* Deletion of estrogen receptor α in skeletal muscle results in impaired contractility in female mice. *J. Appl. Physiol.* **124**, 980–992 (2018).
7. Brooks, S. V & Faulkner, J. A. Contractile Properties of Skeletal Muscles from Young, Adult and Aged Mice. *J. Physiol.* **404**, 71–82 (1988).
